# EXPLAINING THE RACIAL AND ETHNIC DIFFERENCES IN ADL DISABILITY AMONG OLDER ADULTS: A POLYSOCIAL SCORE APPROACH

**DOI:** 10.1093/geroni/igac059.356

**Published:** 2022-12-20

**Authors:** Chenkai Wu, Junhan Tang

**Affiliations:** Duke Kunshan University, Kunshan, Jiangsu, China (People's Republic); Duke Kunshan University, Kunshan, Jiangsu, China (People's Republic)

## Abstract

Disability in activities of daily life (ADL) is prevalent among older Americans. Racial and ethnic disparities in functional ability in old age continue to be a public health concern. We examined whether social environment, measured in a comprehensive way (polysocial score approach), could modify the racial and ethnic differences in ADL disability. Data are from the Health and Retirement Study; 5,925 older adults initially free of disability were included. Six ADLs were considered: bathing, eating, using the toilet, dressing, walking across the room, and getting in/out of bed. We included 24 social factors from five categories (economic stability, neighborhood environment, education, community/social context, and healthcare system) and used forward stepwise regression to screen for important ones. Polysocial score was created using 13 social factors and was classified as low (0-19), intermediate (20-30), and high (31+). We used the multivariable Poisson regression to estimate the risk of incident disability by three polysocial score categories and evaluate the interaction between race/ethnicity (non-Hispanic Whites and Others) and the polysocial score. A higher polysocial score is associated with a lower disability risk among non-Hispanic Whites and Others. We found an additive interaction between race/ethnicity and polysocial score categories. In the low polysocial score group, non-Hispanic Whites had a 4.7% lower risk of disability than the Others, while the difference significantly reduced to 2.4% and 2.6% in the intermediate and high polysocial score group, respectively. The polysocial score approach offers a new opportunity to explain the racial/ethnic disparities in functional capacity among older adults.

